# Editorial: Cognition and Music Performance

**DOI:** 10.3389/fpsyg.2022.923452

**Published:** 2022-06-09

**Authors:** Laura Herrero, Guadalupe López-Íñiguez, Oscar Casanova, Francisco Javier Zarza-Azulgaray, Gary Edward McPherson

**Affiliations:** ^1^Education Department, Camilo José Cela University, Madrid, Spain; ^2^Sibelius Academy, Helsinki, Finland; ^3^Musical, Plastic and Corporal Expression Department, University of Zaragoza, Zaragoza, Spain; ^4^Conservatorium of Music, The University of Melbourne, Parkville, VIC, Australia

**Keywords:** music research, cognition, education, perception, socio-cultural

Because it involves a host of different perceptions, concepts and practices that are linked to particular social considerations, research in music is multifaceted and varied (Cross, 2001). The understanding of music cognition requires the contributions of psychological, neuropsychological, social, cultural, and educational perspectives to provide a full approximation to the phenomenon.

Psychological and neuropsychological studies have reported valuable insights regarding both musical perception and music performance. The cognitive and emotional mechanisms underlying the perception of music and how pitch, time, meter, and harmony are processed are of particular concern to researchers (Justus and Bharucha, 2002), as are how music expectations are produced (Pearce and Wiggins, 2012), how musical imagery is related to music perception (Brodsky et al., 2008; Martin et al., 2018), or how performers can regulate their emotional and motivational processes when preparing for concerts (López-Íñiguez and McPherson, 2020, 2021).

Music performance research has reported on evidence related to motor (Meyer and Palmer, 2003), auditory (Zatorre et al., 2007), cognitive (Jaschke et al., 2018; Herrero and Carriedo, 2019, 2020), and expressive (Canazza et al., 2013) processes during the interpretation of rehearsed or unrehearsed repertoire. In addition, the skills of playing from memory, playing by ear, sight reading and improvising, together with performing rehearsed repertoire are considered the five basic types of solo and group performance (McPherson, 1995a,b).

Whereas music education studies have mainly focused on teaching methodologies (e.g., Pozo et al., 2022), or perceptions about learning (López-Íñiguez and Pozo, 2014; García-Gil et al., 2021), social and cultural research has provided an extension of music considerations by exploring the benefits of music therapy (Gómez-Romero et al., 2017; Shi and Zhang, 2020), analyzing different cross-cultural approximations to music (Cross, 2001), and suppo rting the role of music as a social activity in musicians (Volpe et al., 2016), across the general population (D'Ausilio et al., 2015).

As none of the above-mentioned perspectives work in isolation, the current Research Topic presents a broad approach to music research by encouraging additional novel and diverse contributions.

Thus, musical experiences are addressed from the point of view of performance analysis through physiological and psychological measures focused on whether internal and external parameters are related to performance efficiency. Bojner et al. explore togetherness performance in string quartet by analyzing individual and group “flow” experienced by the musicians. By combining heart rate variability (HRV) and qualitative data, the study shows an interplay of synchronized social interactions among players associated with a feeling of group state flow. Papageorgi explores music performance anxiety (MPA) of adolescent musician learners in order to analyze whether it is interpreted and responded in different situations. The results suggest the existence of three different profiles (low, moderately, and highly anxious students) which are discussed in order to its clinical and educational implications. On their behalf, Orejudo et al. also analyze students of different ages and how the role of social support regarding self-efficacy in learning and performance may be considered as a potential predictor of long-term careers in music. This study shows how as age of performers increases the external social agents related with self-efficacy perceptions is varying. A different approximation performance based on communication is provided by Li et al. who study timbrical expressions and perceptions from the perspective of performers and listeners. Specifically, this study reports whether the timbral intentions of pianists provide visual cues that influence the perceived timbre of listeners. Music performance is also the focus of Tomezzoli et al. who investigate how bow mechanical characteristics influence violinists' preferences. This study shows that both camber and mass impact not only on the appreciations of violinists but also on the evaluations of the experts. Finally, the review of Godøy focuses on the practical applications in performance, improvisation, and composition of the our understanding of generative processes in music through the analysis of the crucial role of the sound-motion objects.

This Research Topic also contributes to increase the evidences in the analysis of educational music performance across cultural contexts. Yaghmour et al. report relevant neuropsychological cues to understand improvisation in a cross-cultural context. Through the analysis of EEG correlates, this study provides the first observations of the tonal-spatial system charactaristic of Middle Eastern music. A different cultural context is explored by Casas-Mas et al.. The authors provide a qualitative-descriptive approach to Flamenco music through the analysis of discourse and practice of two flamenco guitarists in their natural contexts by pointing out the embodied mind as a result of instrumental learning.

Beyond cross-cultural approximations, different social implications of music cognition and performance are also explored in this Research Topic. Neuropsychological evidences using diffusion MRI are reported in the study of Mehrabinejad et al. which reveals that microstructures with functional connection with motor and somatosensory areas as well as language processing area have significant correlation with music engagement and training and that this associations differ by sex. Li et al. contributes to social implications of music performance though the consideration of music to improve the quality of life of people by presenting an extensive bibliographical review about the contributions of music therapy.

Taken together, the papers included in this collection provide a rich insight into current research interested in the relationship between music cognition and performance. Through the use of a wide range of perspectives and methodologies, the articles presented here delve into key issues that allow us to improve our understanding of said relationship as well as its educational and social implications.

## Author Contributions

All authors listed have made a substantial, direct, and intellectual contribution to the work and approved it for publication.

## Conflict of Interest

The authors declare that the research was conducted in the absence of any commercial or financial relationships that could be construed as a potential conflict of interest.

## Publisher's Note

All claims expressed in this article are solely those of the authors and do not necessarily represent those of their affiliated organizations, or those of the publisher, the editors and the reviewers. Any product that may be evaluated in this article, or claim that may be made by its manufacturer, is not guaranteed or endorsed by the publisher.
